# Supplementary material for: Sorting and Manipulation of Human PGC-LC Using PDPN and Hanging Drop Cultures
Source: Cells. 2022 Nov 29;11(23):3832. doi: 10.3390/cells11233832 (PMC9736549; doi:10.3390/cells11233832)
Supplement: Supplementary file 1 [file cells-11-03832-s001.zip › cells-2036621-supplementary.pdf]

Supplementary Figures

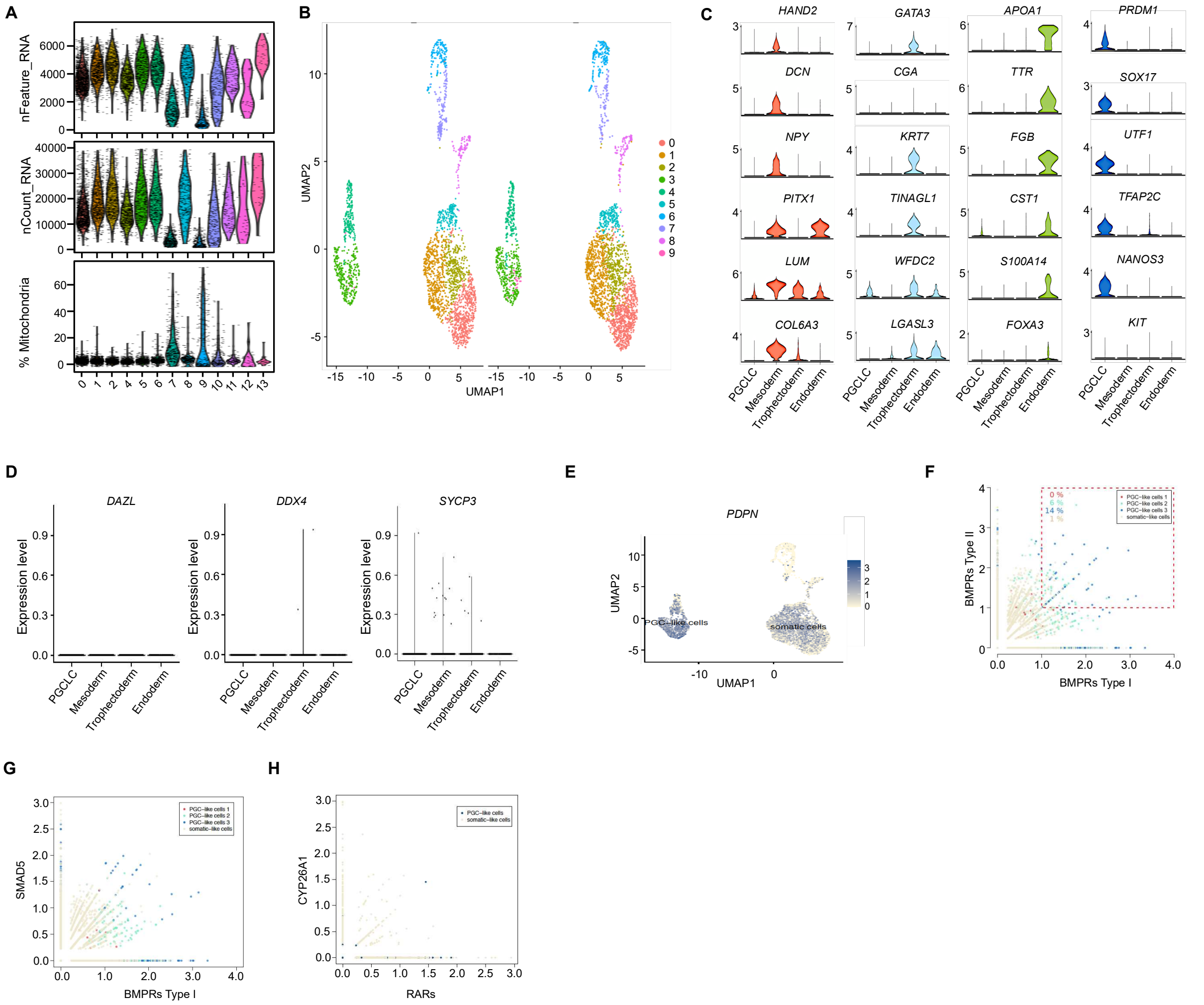

Figure S1. ScRNA-Seq of the naïve iPSC-derived PGC-LCs from day 4 EBs. (**A**) Violin plots showing either the number of genes, UMIs and mitochondrial genes by cells in the indicated clusters. (**B**) Cell clusters identified by the graph-based Louvain algorithm (resolution, 0.8) in scRNA-seq data. Each cluster is defined with a number and labelled with a unique color. (**C**) Violin plots show the expression levels of either mesoderm, endoderm, trophectoderm or PGC marker genes that were used to identify the different cellular lineages present in the day 4 EBs. (**D**) Violin plots show expression levels of late PGC (*DAZL*, *DDX4*) or meiotic (*SYCP3*) cell marker genes. (**E**) UMAP of the integration: points are colored according to the expression level of *PDPN* gene. (**F**) Scatter plot shows the co-expression correlation of Type I *BMPR* with the Type II *BMPR* genes in the indicated conditions. (**G**) Scatter plot shows the co-expression correlation of Type I *BMPR* with the *SMAD5* gene in the indicated conditions. (**H**) Scatter plot shows the correlation of *RAR*s with the *CYP26A1* gene in the indicated conditions.

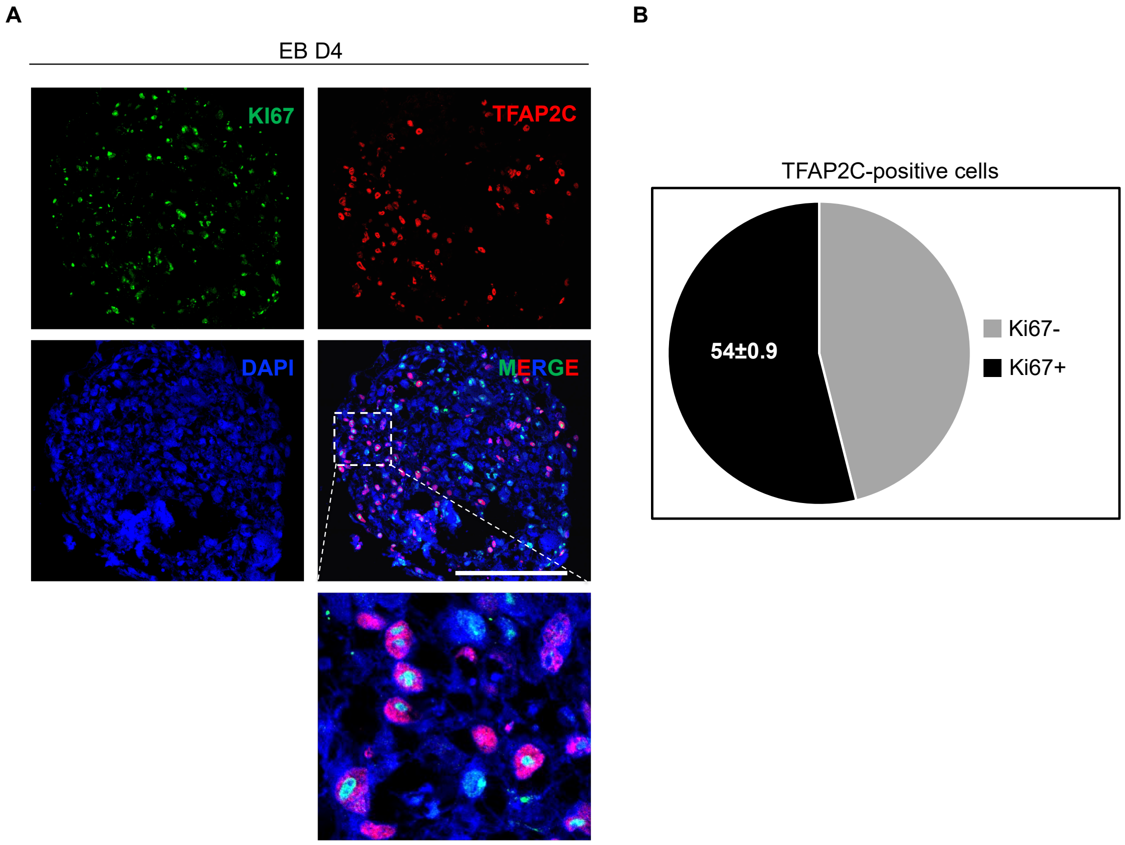

Figure S2. Assessment of early hPGC-LC proliferation potential. (A) Immunodetection of TFAP2C and Ki67 proteins in a day 4 embryoid body. Scale bar, 200 μm. The dotted box is enlarged on the bottom image. (B) The pie chart shows the percentage of Ki67-positive cells within the TFAP2C-positive cells. The results are shown as the mean ± sem, with n=4.

Footnotes

EB D4: Embryoid Body at Day 4.

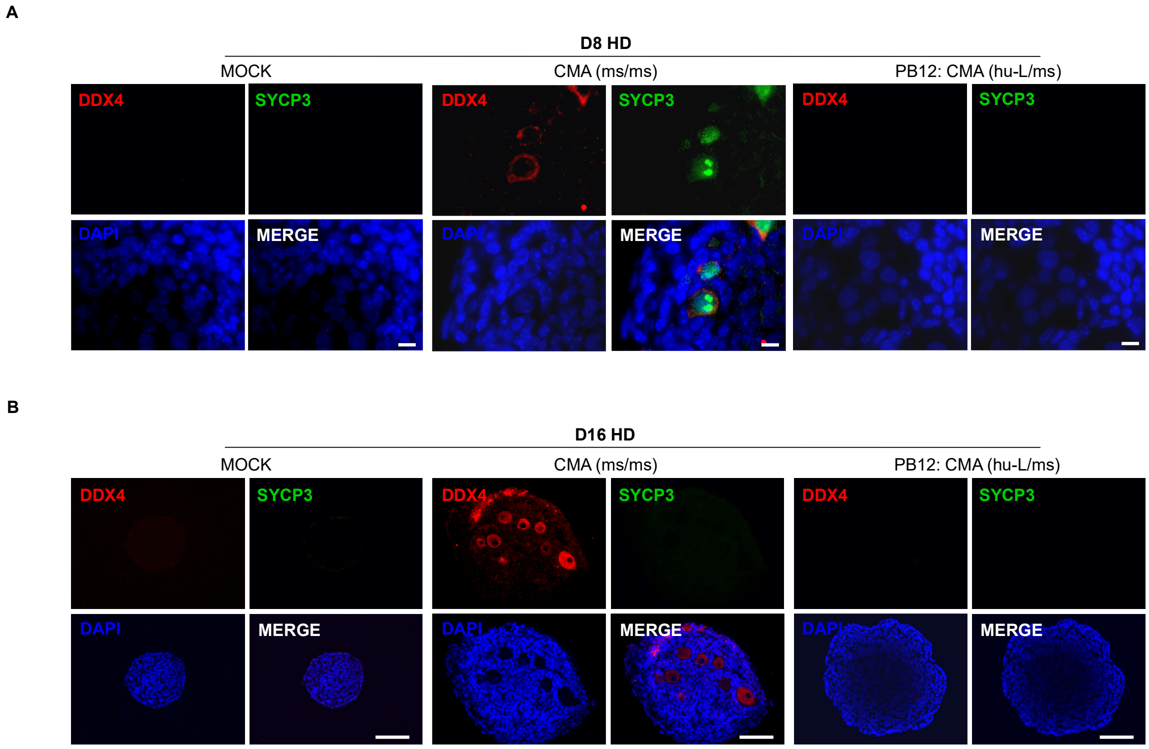

Figure S3. *In vitro* assessment of mouse somatic environment on DDX4 and SYCP3 proteins expression in hPGC-LC-derived from PB12 hiPS cell line. Immunofluorescence analyses for the absence or presence of DDX4 and SYCP3 proteins in MOCK, CMA (Ms/Ms) and CMA (Hu/Ms) conditions at D8 **(A)** or D16 **(B)** of culture. Scale bars, 10 µm or 100 µm, respectively.

Footnotes:

CMA Hu/Ms: Cell Mixture Aggregate (Human PGC-LC/Mouse somatic cells);
CMA Ms/Ms: Cell Mixture Aggregate (Mouse PGC/Mouse somatic cells);
D8 HD: Day 6 from Hanging Drop;
D16 HD: Day 14 from Hanging Drop;
D2 U: Day 2 from U-bottom low adherence 96 well plate;
MOCK: 12.5 dpc ovarian somatic cells only.

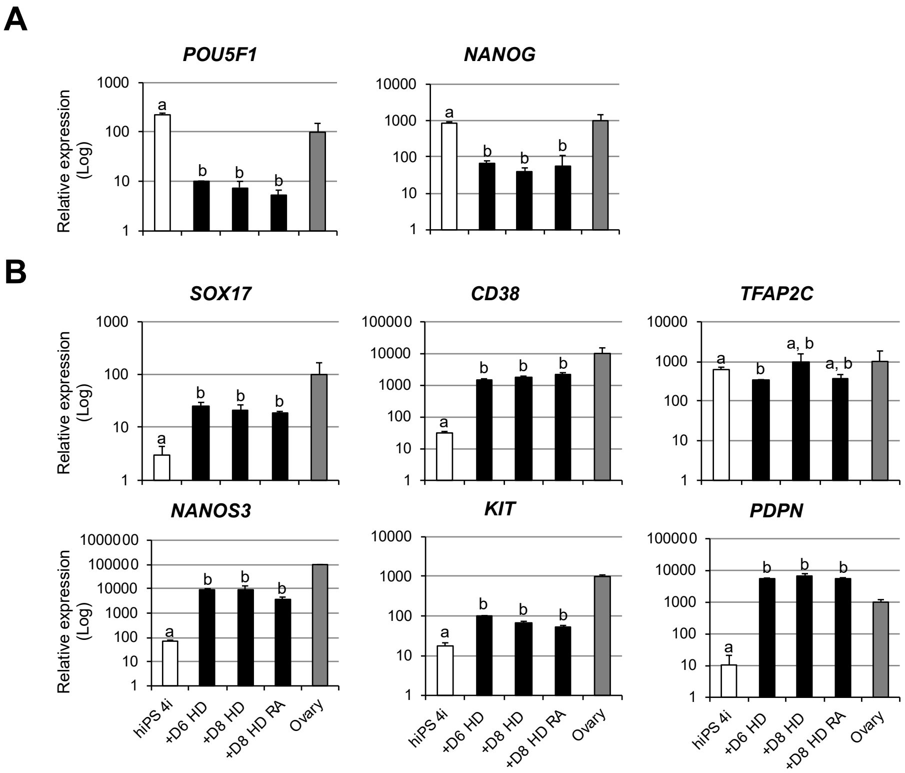

Figure S4. Gene expression analyses of pluripotency (**A**) and early PGC (**B**) markers in hanging drop embryoid bodies. The results are shown as the mean ± sem, with n=4 (4i hiPS) or n=3 (+D6 HD, +D8 HD, +D8 HD RA and human fetal ovary, each). Conditions that do not share a common letter are significantly different ($p<0.05$).

Footnotes:

+D6 HD: +Day 6 in Hanging drop;

+D8 HD: +Day 8 in Hanging drop;

EB D4: Embryoid Body at Day 4.

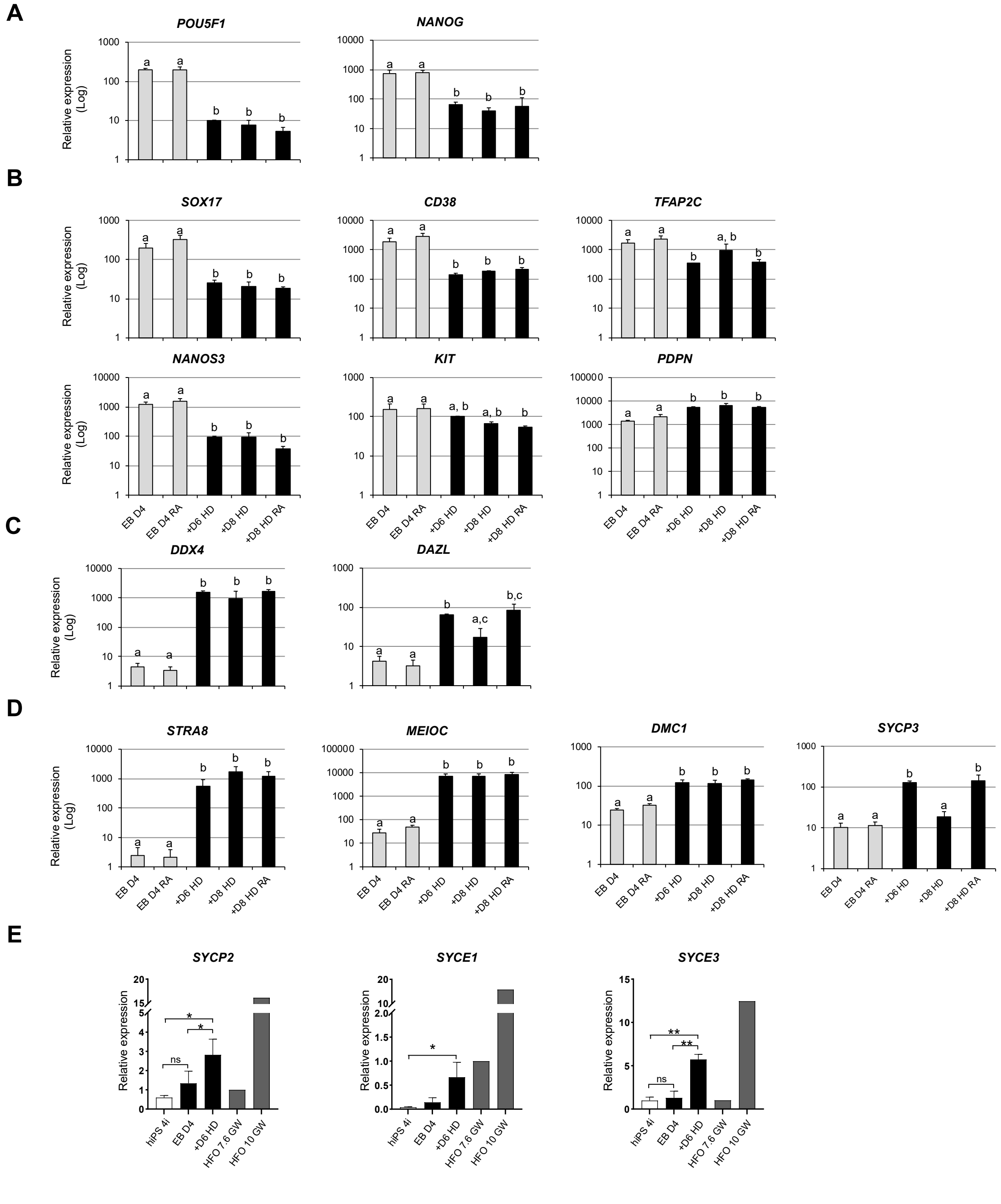

Figure S5. Gene expression analyses of pluripotency (**A**), early PGC (**B**), late PGC (**C**), meiotic (**D**) and synaptonemal complex (**E**) gene markers before and after hanging drop EB culture. The results are shown as the mean ± sem, with n=5 (EB D4), n=6 (EB D4 RA) and n=3 (+D6 HD). In E, gene expression was compared to that in human fetal ovaries (HFO) of 7.6 and 10 gestational weeks and in iPS cell (hiPS 4i), with n=3 except for SYCE1 EBD4, n=2. Asterisks indicate a statistically significant difference (*: $p<0.05$, **: $p<0.01$).

Footnotes:

+D6 HD: +Day 6 in Hanging drop;

EB D4: Embryoid Body at Day 4.

GW: Gestational Weeks

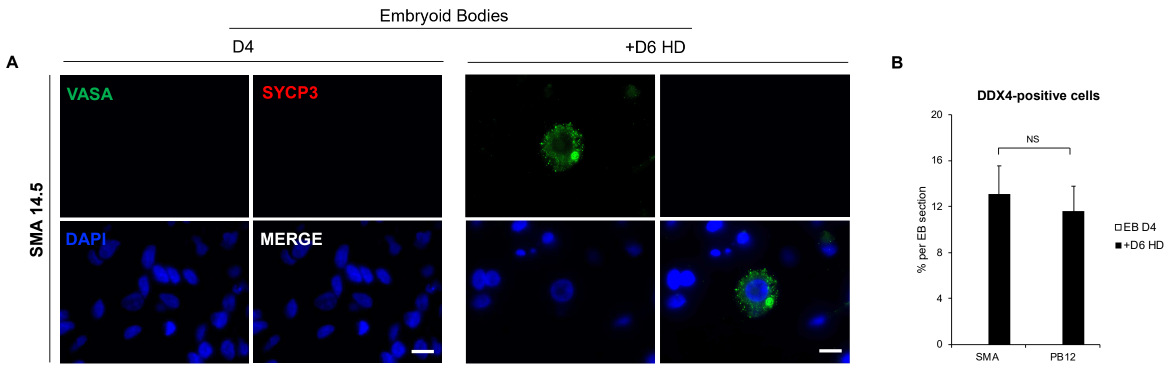

Figure S6. Assessment of late PGC and meiotic proteins expression in SMA hiPSCs-derived EBs at day 4 and day 10. **(A)** Immunofluorescence analyses of DDX4 and SYCP3 proteins expression in SMA hiPSCs-derived EBs at day 4 (left panel) and day 10 (right panel). Scale bar, 10 µm. **(B)** Percentages of DDX4-positive cells per day 4 and day 10 EB section originating from PB12 or SMA hiPSCs. *NS* indicates no statistical difference ($p>0.05$).

Footnotes:

+D6 HD: +Day 6 in Hanging drop;

EB D4: Embryoid Body at Day 4.

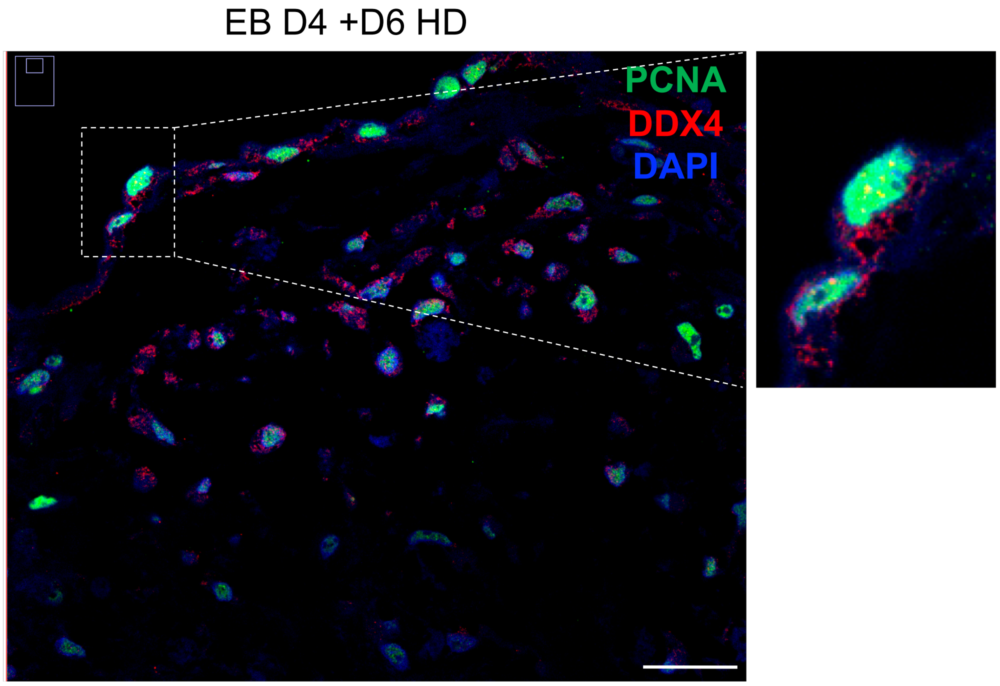

Figure S7. Assessment of late hPGC-LC proliferation potential. Immunodetection of DDX4 and PCNA proteins in hanging drop-cultured embryoid bodies at day 6. Scale bar, 50 µm. The dotted box is enlarged on the right image.

Footnotes:

+D6 HD: +Day 6 in Hanging drop;

EB D4: Embryoid Body at Day 4

Supplementary Table S1. List of primers used for RT-qPCR.

List of the targeted genes		
**Gene**	**Forward (5' to 3')**	**Reverse (5' to 3')**
ACTB	TGACCCAGATCATGTTTGAGA	TACGGCCAGAGGCGTACAGG
CD38	GGCGCGATGCGTCAAGTACACTG	CCTAGCAGCGTGTCCTCCAGGGTG
DAZL	TACTCCACCCTCTGGAAATGG	GCTTCGGTCCACAGATTTCTT
DMC1	GGGAGACTGTGG GTACGAGG	AAAGTGGGCAACAGAAAAATAATCA
KIT	TGAATCTACTTGGAGCCTGCAC	CTGTAATGACCAGGGTGGGC
MEIOC	ATCGGCAAAGGCAAGGAGT	GCGTGTTTTCCGAGTAGCCA
NANOG	TGGCCGAAGAATAGCAATGG	AGTCGGGTTCACCAGGCAT
NANOS3	AGGAGCAGGTTTCAGAGGTGC	AGAGCAGGAGGGCGAAGG
POU5F1	CTGCAGCAGATCAGCCACAT	CACATCCTTCTCGAGCCCAA
PDPN	TGTGGTTATGCGFAAAAATGTCG	CCTTCAGCTCTTTAGGGCGAG
SOX17	GAGCCAAGGGCGAGTCCCGTA	CCTTCCACGACTTGCCCAGCAT
STRA8	CTGGACAAAAGTGAGGTTCCG	GGCAAGCACTGAACTGGAGC
SYCP3	GAGCCTATGACTTTGAGACTG	CAACTACTCCTGCAGAAGAC
TFAP2C	GTACGAAGAGGACTGCGAGGA	GGATTCCCATTGCTGCTCC
DDX4	GCCTCTGGGCGGAATTTT	CGCTTATTACACTCACCAGCATG
ACTB	GACCCAGATCATGTTTGAGA	TACGGCCAGAGGCGTACAGG
SYCP2	AGCGAACAACAGAGGCTTCA	AGCATGTCCTTAAGAAGCCTGT
SYCE1	GCACACAAGAGGAAGAGGCT	TGTGTCCTCCTGGCCTATGA
SYCE3	TGGATGATGCTGACCCTGAG	CACCACCATGTCATAGGCCA

Supplementary Table S2. List of antibodies used for Immunohistochemistry or immunofluorescence.

List of the antibodies used in the study						
**Antibodies**	**Company**	**Reference**	**Host specie**	**Mono/Polyclonal**	**Application**	**Concentration**
DDX4	Abcam	ab27591	Mouse	M	IHC/IF	1/200
DDX4	Abcam	ab13840	Rabbit	P	IF	1/200
POU5F1 (OCT-3/4)	Santa Cruz	sc-5279	Mouse	M	IHC	1/50
PDPN (D2-40)	DAKO	M3619	Mouse	M	IHC	1/20
SYCP3	Abcam	ab97672	Mouse	M	IF	1/500
SYCP3	Novus	NB300-232	Rabbit	P	IF	1/400
TFAP2C	Santa Cruz	sc-12762	Mouse	M	IHC/IF	1/200
KI-67	Novus	NB110-89717	Rabbit	P	IF	1/200
Alexa 488 anti-mouse	Thermofisher	A21202	Donkey	P	IF	1/500
Alexa 594 anti-rabbit	Thermofisher	A21207	Donkey	P	IF	1/500
Alexa 488 anti-rabbit	Thermofisher	A21206	Donkey	P	IF	1/500
Alexa 594 anti-mouse	Thermofisher	A21203	Donkey	P	IF	1/500

Footnotes:

IHC: Immunohistochemistry;
IF: Immunofluorescence;
M: Monoclonal;
P: Polyclonal.
